# Sustaining biological welfare for our future through consistent science

**DOI:** 10.1186/1880-6805-32-1

**Published:** 2013-01-15

**Authors:** Yoshihiro Shimomura, Tetsuo Katsuura

**Affiliations:** 1 Graduate School of Engineering, Chiba University, Chiba, Japan

**Keywords:** Biological benefit, Ergonomics, Inconsistency, Integrative thinking, Physiological function, Universal design

## Abstract

Physiological anthropology presently covers a very broad range of human knowledge and engineering technologies. This study reviews scientific inconsistencies within a variety of areas: sitting posture; negative air ions; oxygen inhalation; alpha brain waves induced by music and ultrasound; 1/*f* fluctuations; the evaluation of feelings using surface electroencephalography; Kansei; universal design; and anti-stress issues. We found that the inconsistencies within these areas indicate the importance of integrative thinking and the need to maintain the perspective on the biological benefit to humanity. Analytical science divides human physiological functions into discrete details, although individuals comprise a unified collection of whole-body functions. Such disparate considerations contribute to the misunderstanding of physiological functions and the misevaluation of positive and negative values for humankind. Research related to human health will, in future, depend on the concept of maintaining physiological functions based on consistent science and on sustaining human health to maintain biological welfare in future generations.

## Review

Physiological anthropology encompasses the fields of what is good and comfortable in the design of tools, environments, or systems based on human characteristics, in order to increase the quality of life. The perspective of physiological anthropology, in terms of research methodology, is much broader than that of other research disciplines. For example, a ‘human being’ has many meanings in human science, such as a pure biological entity [1], an anthropological entity that progressively forms cultures and societies [2], a maker or user of artificial materials or environments [3,4], and a task-based entity, such as that discussed by Rasmussen [5]. However, all aspects of previous research regard humankind scientifically, and researchers believe that analytical methodology is the way to categorize humans in discrete detail, even though an individual comprises a unified set of whole-body functions. Brewer and Hsiang [6] described multidisciplinary human science and engineering, such as ergonomics, as targeting future challenges. They consider that methodological unification and its expansion are important. Boff [7] described revolutions and shifting paradigms of ergonomics over four generations, in fields ranging from the workplace and biomechanical capability to the biological enhancement of physical or cognitive capabilities. The emerging ethical problem that they highlight is the relationship between human beings and technology, and they warn us of the importance of optimal integration. On the other hand, the technological adaptability and physiological polymorphism described by Sato [8] and its application to manufacturing and design [9] might provide researchers with a basic concept for considering how human beings, who have evolved over a period of five million years, should adapt to the explosive development of today’s technological environment. Although some studies, such as these conceptual frameworks, have attempted to lead future human science, the philosophy of science for the welfare of humanity remains obscure because the word ‘human’ remains undefined and the concept of the ‘human’ varies between researchers. Therefore, this present study reviews scientific inconsistencies resulting from analytical considerations of technology and human beings, and discusses these issues from various perspectives to advance interpretations based on integrative thinking. The items discussed in this study refer to keywords in scientific design considerations in physiological anthropology [10], and were selected to contain a variety of areas of human science, such as the musculoskeletal, circulatory, and central nervous systems, brain function, and stress relief. A concept for today’s human technological lifestyle is proposed from the perspective of sustained biological welfare for future generations.

### Is there a consistent understanding of ‘human’?

Although all previous studies of human science and engineering with a proper purpose and valid experimental techniques must be correct in terms of analytical science and a single understanding of what ‘human’ means, inconsistencies have arisen. These examples help to clarify these scientific inconsistencies and provide a platform for discussion.

### Sitting posture for video display terminal workers

**Figure 1:**
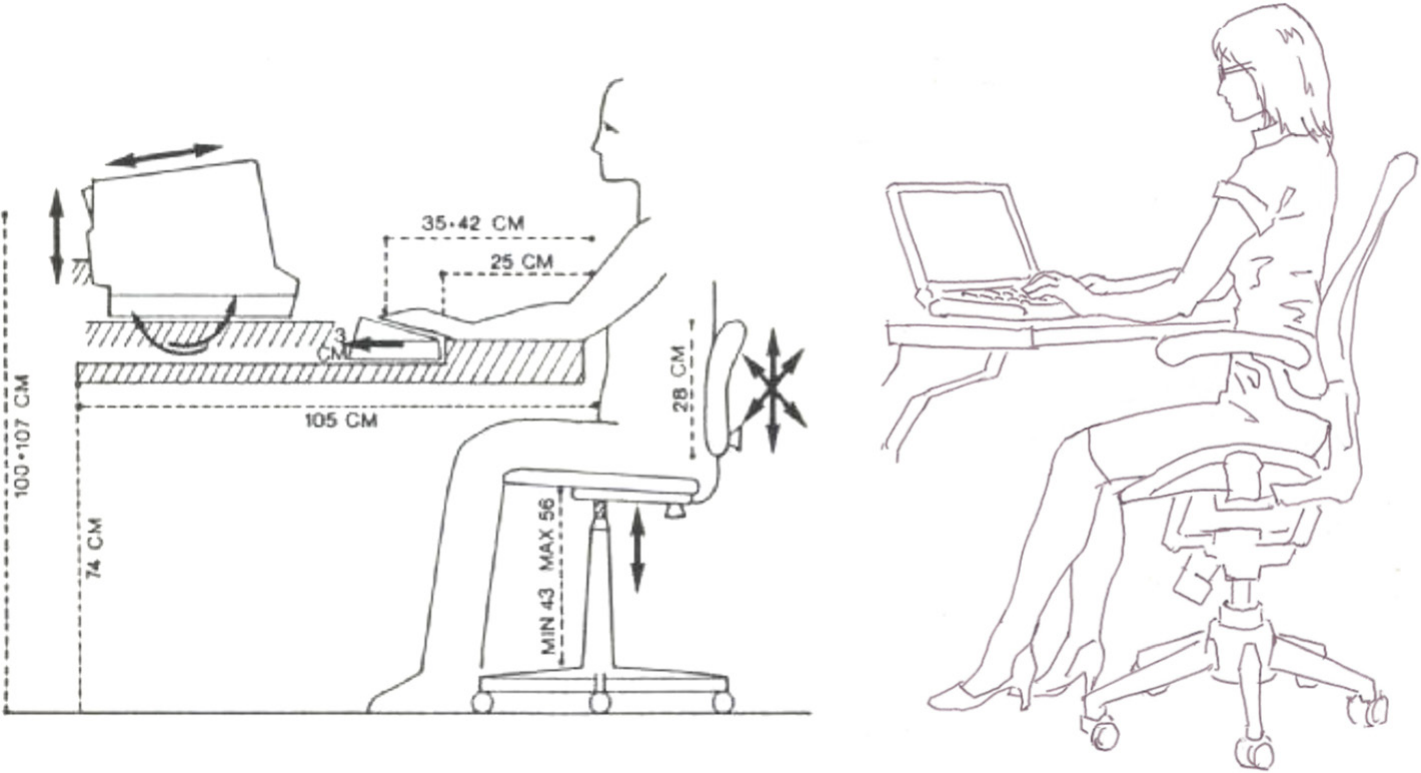
**The proper sitting posture commonly recommended by researchers. **The picture on the left was drawn by Grieco [71] and that on the right shows that recent trends have not changed at all since then.

To fit the height of a chair to a human leg and lean the head downward a little is held to be common-sense ergonomics for video display terminal (VDT) workers [11-13] (Figure 1). However, a horizontal seat pan forms a smaller angle between the spinal column and the thigh, and is thus associated with an unequal distribution of tension in the ligaments and muscles of the anterior and posterior sides of the spine [14,15] because of post-bending the spinal column [16-18]. Although some declination of the head might help to avoid eyestrain, the shoulders and neck must stiffen to support the weight of the head (about 5 kg) via steady-state activation of the trapezius and neck extensor muscles [19,20]. Since the earliest phase of ergonomics, no researcher has been able to design the most appropriate chair. The problem of designing a chair is a typical example of the issues that require integrative thinking about physiological functions of the total body, such as overall spine biomechanics, blood flow, leg edema, digestion, and cognitive function.

### Negative air ions

Many reports have scientifically explained the comfort gained from being near a waterfall or a forest where negative ions are in the air. Some have demonstrated that negative air ions reduce physiological and psychological stress and exert positive effects on human beings [21-23]. In contrast, others contradict these findings [24,25]. One study even demonstrated that electrically generated negative ions in air can cause pathological damage [26].

Thinking rationally, molecular clusters of water [27] are not large enough to be absorbed through the pulmonary alveoli. The reduction in stress markers might simply have been caused by humidity generated by water protecting the skin and mucosa, along with possible negative ions in the air [28].

### Oxygen inhalation

‘Oxygen bars’ have recently become popular as thought is supposedly clarified, and recovery from fatigue is rapid. However, the typical arterial oxygen saturation of a healthy individual is just above 95% [29]. Moreover, measurements of blood flow in the cerebral cortex demonstrate that astrocytes control the functional hyperemia and result in sufficient oxygen to match the amount of consumption [30].

It is, in fact, not known whether an excess of oxygen exceeds the balance of homeostasis in blood. Superfluous and long-term oxygen inhalation might increase the production of free radicals [31], and might be deleterious to health [32,33]. Researchers must consider not only the immediate effects of the technology but also the long-term effect of its continuous application.

### Alpha waves induced by relaxation music

Many types of music might increase the quantity of alpha brain waves, as measured in electroencephalograms. Iwaki *et al.*[34] showed an increase in alpha band amplitude with music stimulation, while Kawasaki *et al*. [35] clarified that music-induced pleasure increases the intensity of the alpha waves. Many reports have indicated that the production of alpha waves is increased by relaxation.

Incidentally, one study has shown that rates of alpha waves increased more during an experiment with relaxation audio stimuli than with a general music. However, the subject of that experiment had closed his eyes as the relaxation audio was played. This was reflected as changes in brain activity with or without vision-processing [36]. Because human beings are the sum of a total of many body functions, to isolate and consider only a single part of the whole is treacherous. Physiological indexes should be correctly interpreted.

### Ultrasound can be perceived

Ultrasound found in nature, wind chimes, and gamelan music is generally thought to induce relaxation with an increase of the alpha-wave activity [37-39]. However, an auditory brainstem response to ultrasonic tone bursts at a suitable amplitude modulation showed that the sound does not reach the brain stem [40]; that is, humans cannot perceive ultrasonic sound.

Ultrasonic vibration signals delivered as experimental sound stimuli through speakers might lead to slight distortion [41] within the audible range [42], and possibly generate a different sound. The use of more precise technology to evaluate human perception should resolve this inconsistency.

### 1/*f* fluctuations

The vibration or periodic phenomenon in which amplitude or power is inversely proportional to frequency, especially in logarithmic scales, is defined as 1/*f* noise [43]. High- and low-frequency vibrations should have a small and a large amplitude, respectively, and the amplitude should be infinite at a direct current content of 0 Hz, if energy equilibrium is assumed. The 1/*f* property is a very appropriate and convenient means to explain natural periodic phenomena in statistical mechanics.

Biological parameters, such as heart rate and electroencephalograms, follow 1/*f* fluctuations [44-46]. However, respiratory sinus arrhythmia [47,48] is absent if heart rate variability maintains 1/*f* scaling. Furthermore, 1/*f* properties do not show dominant bands, such as alpha waves, in electroencephalograms [49]. Living things, from microbes to human, control statistical probability by coordinating their organs to complete themselves. Therefore, inductive thinking based on statistical equivalence might not be the most appropriate way to explain the whole body of a living thing.

### Evaluation of feelings using surface electroencephalography

Several reports have discussed the determination of feelings and emotions using electroencephalography [50-52]. According to the somatic-marker hypothesis [53], emotion is the result of a series of changes in body states, such as the defense and stress reaction, in which the amygdala processes input signals from whole-body receptors. The hypothalamus, hypophysis, and brain stem in the deeper part of brain control these body states.

Native emotions, such as fear and joy, are adaptive systems automated by the limbic system and the Papez circuit [54,55], whereas the frontal lobe and ventral medial field change physiological states through native emotion circuits to express more complex acquired emotions, such as sympathy, jealousy, or pleasure. The insular cortex and the somatosensory cortex area perceive body reactions in emotions, and then the frontal area processes information and builds feelings based on individual experience and memory. Feelings are induced through such multi-regional coordinated processes. Thus, measuring feelings with only a surface electrode is theoretically impossible. The limitations of a methodology should be strictly understood by researchers, if they are to form a valid description of what is ‘human’.

### Kansei

Kansei is the ability to recognize by intuition [8]. This ability is cultivated by bypassing the perception of a somatic reaction [53] by the frontal lobe, such as the orbitofrontal cortex via the insular cortex and other limbic regions [56,57]. Somatic reactions are marked by the frontal lobe as positive or negative, and the internal end point can be judged immediately without any intervening stage or conscious use of reasoning [8]. Decision-making by an individual is not always rational and logical, but can be illogical and intuitive. Linguistic explanations and social thinking occur thereafter. Therefore, subjective evaluation that can be described in symbols does not express Kansei. Studies of Kansei engineering [58-60] might not have necessarily evaluated Kansei appropriately.

In addition, the range of the Kansei reach is restricted within individual experience and is very narrow, which is a drawback for designers.

### Universal design

Currently, universal design is the adaptation of a product or environment to various users by involving a group that is challenged by some obstacle or by age [61-63]. This is the same as improving usability and accessibility for a classified user group.

Nominally, human beings have a large brain, dexterity, and the ability to walk. However, morphological, functional, and cultural aspects vary considerably among worldwide populations. Essential discussions about what is universal for human beings, the nature of design and what it is to be human do not exist in the current literature. This condition is insufficient for the establishment of a ‘universal’ science.

### Anti-stress

**Figure 2:**
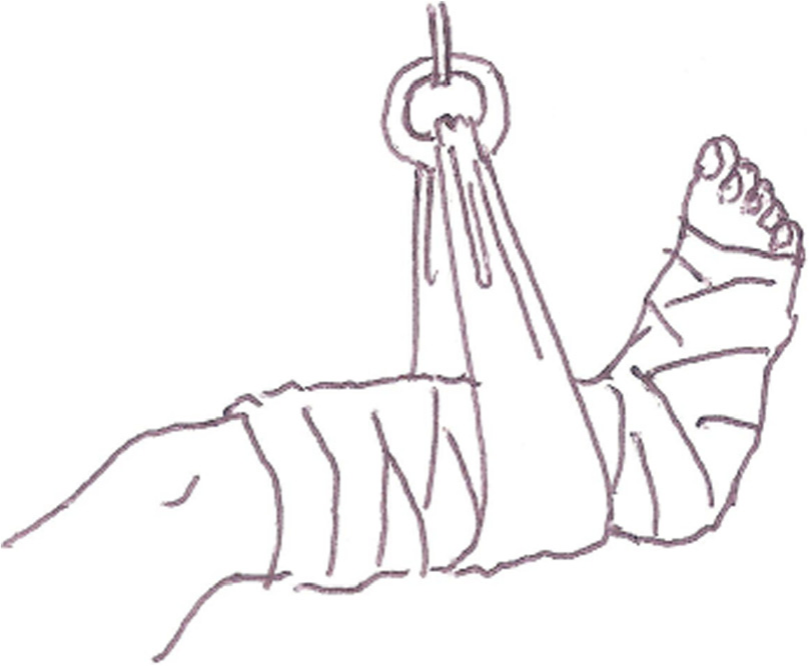
Symbol of a quiet state.

The reduction of physiological stress is very important in many of today’s human sciences, such as ergonomics [64-67]. However, humanity cannot maintain mental and physiological functions without stress. All current industrial products and environmental designs have focused on suppressing stress in human beings to achieve comfort and to remove inconveniences or difficulties from the environment. Technology has now reached a point where disuse atrophy (Figure 2), potential reduction, and functional degeneration are significantly promoted. Stress is simply a reaction to a stressor, so good and bad stress originally had no meaning. Stress should be carefully addressed by integrative thinking, especially in physiological anthropology, rather than based solely on research from individual disciplines.

### Expectations for consistent science

Scientific inconsistencies caution that human beings should not be interpreted from a single intelligible analytical perspective. Intelligible analytical science will deprive humanity of the opportunity for integrative thought [68-70]. Thus, if arguments range across various disciplines, inconsistencies will frequently arise, even if each study is appropriate in terms of analytical details. The perspective of physiological anthropology is much wider than that of other research disciplines, so it essentially has an interdisciplinary methodology. Physiological anthropologists should not be satisfied with only an analytical way of science.

As noted by Sato [8], modern human beings evolved through harsh environmental conditions over a period of about five million years. All human physiological functions were created solely to adapt to nature. Today’s human sciences appear simply to detail observations of human beings in terms of only single aspects of evolutionary outcomes and might forget that human beings have a long history of adapting to nature. Therefore, researchers should think not only about the phenomenon currently facing them but also of the physiological functions that comprise the total human being.

To maintain present conditions and quality of life would be impossible without the support of life engineering, antibiotic, and medical technologies. For human science to progress under such circumstances, the concept of maintaining physiological functions and expanding biological adaptation ability by suitable stress management should perhaps be based on consistent science. In addition, the health of *Homo sapiens* should be sustained to establish a biological welfare that will persist for generations to come.

Human beings have invented various devices and created tools and viable environments over five million years of evolution. This process is an act of design that is unique to humankind and it mostly engages integrative thinking. Physiological anthropology could also be considered as the discipline for developing the human act of design. A significant argument regarding the future of physiological anthropology will hopefully be generated with respect to integrative scientific research and a steadfast vision of the future of humanity based on current technological lifestyles.

## Conclusion

Scientific inconsistencies warn of the importance of integrative thinking and the biological benefit to humanity. We propose that physiological functions based on consistent science should be considered so that health, or biological welfare, can be sustained over the long term.

## Abbreviation

VDT: video display terminal.

## Competing interests

The authors declare that they have no competing interests.

## Authors' contributions

YS conceived and wrote the manuscript. TK conceived and helped to draft the manuscript. Both authors have read and approved the final manuscript.
